# Enhancing the quality of clinical trials in cancer pain first requires that pain assessment methods are appropriately understood and used

**DOI:** 10.1038/sj.bjc.6603357

**Published:** 2006-09-26

**Authors:** A Caraceni, C Brunelli, C Martini, E Zecca, F De Conno

**Affiliations:** 1 ‘Virgilio Floriani’ Hospice and Palliative Care Unit, National Cancer Institute of Milan, Via Venezian 1, Milan 20133, Italy

**Sir**,

We read with interest the article by Bell *et al* (2006) that reviewed the quality of the methodology in cancer pain clinical trials. It has also been our experience that the methodology employed in these trials has frequently been less than optimal. To further quantify this problem, we undertook a systematic qualitative review that specifically explored the pain assessment methods used in oncological clinical trials (Caraceni *et al*, 2005) and reached similar conclusions to those of Bell and colleagues.

For our review, we examined 68 journal articles published between 1999 and 2002 that had chronic cancer pain as the primary or secondary objective of a controlled clinical trial. Only 57% of these studies reported on the clinical characteristics of the cancer pain, and in 81% pain pathophysiology was not mentioned.

More specifically, regarding the use of pain assessment methods, we observed that:

- 10% of the studies used nonvalidated pain measurement methods;
- 70% did not state the time frame over which the pain experience was assessed;
- the method of administration (e.g. self-administered or external observer interview) of the pain measurement tool was not described in 46% of studies;
- a clear definition of the pain outcome measure was missing in 40% of studies;
- compliance with pain assessment was reported in only one of the studies;
- only 25% of the studies gave a detailed description of the missing data, another 44% provided a rough description of the technique employed to deal with such data, and 31% made no mention of this problem at all.

Our review concluded with a number of recommendations that we believe will enable researchers and clinicians to approach this field of study with more consistency and rigor – through an enhanced understanding of how to choose, implement, analyse and report pain measurement methods and data in oncology clinical trials.
